# Ten quick tips to perform meaningful and reproducible molecular docking calculations

**DOI:** 10.1371/journal.pcbi.1013030

**Published:** 2025-05-09

**Authors:** Elvis A. F. Martis, Stéphane Téletchéa

**Affiliations:** Nantes Université, CNRS, US2B, UMR 6286, Nantes, France; SIB Swiss Institute of Bioinformatics, SWITZERLAND

## Abstract

Molecular docking is a useful method for predicting the binding affinity and conformation of small chemical entities to support lead optimisation. It is also used to virtually screen a large chemical database to find new chemical entities. There are several docking programs available with different algorithms and varying preparation steps. We identify ten quick tips that apply to molecular docking irrespective of the program one might choose. Our objective is to provide the beginners with important things to keep in mind while using molecular docking for their research. We aim to ensure that experts and beginners can perform molecular docking to yield biologically relevant and reproducible results.

## Author summary

The ten quick tips presented here are aimed at understanding the drug target thoroughly and performing molecular docking to ensure maximum precision and biological relevance. The emphasis is not on blindly trusting the results, but on thoroughly validating every step and parameter used in the docking programme. We aim to make beginners and experts aware of all the steps one needs to take care about to ensure reproducibility of the results. Further, these tips highlight the key areas to keep in mind while communicating the results to experts as well as non-experts without ambiguity.

## Introduction

Drug discovery [1] is an arduous journey with many unknowns all along the path, with several traps that lead to failure. To perform large number of trials in an iterative manner, computer simulations [2, 3] come in handy to perform tens of thousands of calculations to provide crucial insights and rule out dead ends. Structure-based drug discovery (SBDD) is one such approach that aids in designing and optimizing chemical entities by leveraging the tertiary structural information of biological targets. SBDD encompasses various strategies, including virtual screening, where large libraries of compounds are screened for its binding affinity with the target site. This process is also called as “hit identification”, where hits are referred to as molecules that show weak yet measurable binding affinity. Hits generally contain most of the chemical framework capable of showing the desired pharmacological response, however, they need optimisation to improve the binding affinity, and other physicochemical properties essential for developing a clinical candidate. Most of the docking programs have shown great success at the lead modification stage, which involves evaluating the effect of the new substituents on the same central core (also called a parent nucleus) [4]. Another important method, called as *de novo* drug design, involves constructing new molecules from molecular fragments within the binding site of the target. Molecular docking [5] is an important technique in structure-based drug discovery (SBDD) that predicts optimal binding conformation of a small molecule (ligand) within the binding site of a biological target. This process helps identify potential drug candidates by estimating their binding affinity and types of interactions to receptor atoms. The latter information is used to suggest modifications in the hit molecules, such that their binding affinity is improved. Iterative cycles of design, synthesis, and experimental validation refine these compounds to optimize their pharmacological properties, this process is called as “lead optimisation”.

Molecular docking has been used for more than four decades to attain two primary objectives:

- To predict the binding affinity and conformation of small molecules within a receptor site, and
- To identify hits from a large chemical database to search for diverse chemical scaffolds [6].

Despite classifying these objectives behind molecular docking into separate classes, their boundaries are not clearly demarcated. To attain these objectives, molecular docking programs must comprise components that can search the conformational space within the binding site and a scoring function that scores the conformations such that the biological relevant ones are ranked higher.

### Conformational search methods

One of the most challenging aspect of pose prediction is the conformational search algorithm. In general, there are two main classes of conformational search methods, systematic and stochastic. The users do not always have a choice to select the search algorithm because the developers usually select based on their expertise and code development. Nevertheless, there are programs, such as AutoDock [7], that allow users to select the search algorithm. Here, we succinctly discuss commonly used conformational search methods. However, this list is far from being comprehensive, interested readers are directed to consult the following papers for detailed discussion [8, 9].

#### Systematic methods.

These methods thoroughly explore all potential conformations exhaustively by systematically changing one or more torsional degrees of freedom. These degrees of freedom arise from the single bonds that can be rotated to attain different atomic orientation with respect to each other (henceforth called as â€œrotatable bondsâ€#157;). We present a brief outline of the methods that fall within the realm of systematic search methods.

- *Systematic Search*: The systematic search algorithm [10] rotates all possible rotatable bonds (single bonds in the ring structures cannot be rotated as freely as open-chain structures) systematically by a fixed interval to explore all possible conformations. Such a method can explore all possible conformations, however, the complexity increases exponentially as the number of rotatable bonds increases. There are pruning algorithms that act as a â€œbump checkâ€#157; that rule out torsion angles that lead to significant atomic overlaps. Furthermore, docking algorithms employ the search algorithm within the binding pocket, and hence, more rotations will be pruned out that may lead to atomic overlaps with receptor atoms. Glide [11] and FRED [12] are examples of two docking programs that employ this method for conformation search.
- *Incremental Construction*: In this method [8], the molecule is broken down into individual fragments. The fragments are then docked in the most suitable sub-pocket in the binding site. The entire molecule is then sequentially built by adding the linkers. This follows a systematic search for the linker conformation that optimally holds the fragments in their respective sub-pockets. The fragmentation method selects the rigid molecular components, for example ring structures, as fragments, and the flexible components as linkers. In doing so, this method reduces the computational complexity compared to the systematic search by focusing on small components, more particularly the linkers. FlexX [13] and DOCK [14] are examples of the docking algorithms that employ this method as its conformational search algorithm.

#### Stochastic methods.

Stochastic techniques utilize random sampling and probabilistic methods to explore the conformational space. They work by randomly making changes, preferably, to the rotatable bonds and evaluates the energy following the change. Metropolis Monte Carlo, and Genetic algorithm are popular stochastic search methods that are used as conformational search tools in docking programs.

- *Monte Carlo*: It works on the principle of random sampling for a predetermined number of iterations to locate optimum conformation. The search algorithm starts by making random changes in any of randomly selected rotatable bond. Following which, it evaluates the energy and compares it with the starting conformation. If the energy of the new state is lower than the previous state, then the conformation is accepted. If the energy of the new state is higher than the previous state, then it assigns a random number between 0 and 1 and evaluates its probability against the Boltzmann distribution; if the random number assigned is greater than the probability, then the new state is accepted otherwise rejected. In case of the rejection, the algorithm restarts from the previous conformations. Glide [11] docking program is also known to employ Monte Carlo simulation in its docking algorithm to improve its pose prediction accuracy.
- *Genetic Algorithm (GA)*: This method [15, 16] is motivated from the process of natural selection, that evaluates energy or docking score as the fitness criteria for selection and breeding. The conformational degrees of freedom are encoded as binary numbers, similar to natural selection, which represent specific values of the torsion angles. At the beginning, several random mutations are made in the binary numbers (interchanging â€œ0â€#157; to â€œ1â€#157;) and generating an initial population. The fitness score is evaluated for each member. Following which the top ranked (fittest members) are retained for breeding. To generate future generations, either cross-over is performed or mutations. This process is repeated for a pre-determined number of iterations. Autodock [7] and GOLD [15] are examples of docking programs that use genetic algorithm as their conformational search tool.

### Molecular dynamics simulations

At this point, it is appropriate to gently introduce another powerful conformational search method, namely *molecular dynamics (MD) simulations*. It simulates the atomic motions as a function of time by solving the Newton’s equation of motion [17]. Molecular docking and MD simulations are complementary methods commonly used in structure-based drug discovery to understand protein-ligand interactions and their dynamics [18, 19]. For computational efficiency, molecular docking algorithms treat receptors atoms rigid and the ligand as flexible. This leads to incorrect pose prediction when induced fit binding is observed. Therefore, MD simulation is an elegant method to incorporate such induced fit effects. This can be achieved in two ways, as shown below:

- Pre-docking step to sample various conformations of the receptor without the influence of the ligand. This can serve as multiple receptor conformations for docking.
- Post-docking step to refine the docked receptor-ligand conformation by allowing the complex to evolve to their realistic conformation, which would be more probable physiologically.

### Implementation of artificial intelligence in molecular docking

With a near exponential increase in the tertiary structure information in the last 3 years attributed to several artificial intelligence based methods, particularly Alphafold [20] and RoseTTAFold [21], many efforts have been made to incorporate machine learning methods in drug discovery to improve the quality of therapeutic agents and reduce the drug discovery timelines significantly [22]. Developers are also constantly developing methods that utilize machine learning techniques to enhance the accuracy of molecular docking predictions. Incorporating the state-of-the-art deep learning neural networks have shown to improve conformational search algorithms and develop better and more generalised scoring functions. These improvements have helped ameliorate challenges posed by limited structural and small molecule data [23, 24]. AI techniques enhance traditional molecular docking methods by introducing innovative strategies such as network-based sampling [25] and unsupervised pre-training [26, 27]. AI-Bind [28], for instance, combines network science with unsupervised learning to mitigate issues like over-fitting and annotation imbalance. It identifies negative protein-ligand pairs using shortest path distances and learns node feature representations without relying heavily on limited binding data. By utilizing node embeddings from extensive chemical and protein structure collections, AI-Bind captures a broader range of structural patterns. Additionally, models like IGModel [29] leverage geometric graph neural networks to incorporate spatial features of interacting atoms, improving binding pocket descriptions. AI-Bind can also predict active binding sites from amino acid sequences alone, without needing 3D structures. Overall, these AI-driven approaches significantly improve the accuracy and generalization of predicting protein-ligand interactions, representing a major advancement over traditional methods.

### Scoring functions

Scoring functions are designed to reproduce the binding thermodynamics [30, 31], *i.e.*, binding enthalpy and entropy (see Eq 1).

$$\;\Delta G_{binding}=\Delta H-T\Delta S$$ (1)

Where ΔH is the enthalpy component of binding and TΔS the entropy component of binding at temperature “*T*”. The enthalpy of binding can be estimated by the number and type of interactions between the receptor and ligand at the atomistic level. Hence, scoring functions often estimate the enthalpy component by summing all interactions of different types. However, this is a non-trivial task, which has also been criticised for being treated as purely additive [32, 33]. Nevertheless, different types of scoring functions employ different methods to fit experimental binding data to develop scoring functions. This leads to different types of scoring functions that can be classified into four types. We briefly discuss the scoring functions and their types, for more detailed discussions the readers are directed to consults these papers [34, 35].

For a docking tool to be effective and yield reliable result, the search algorithm and the scoring function are crucial and carefully developed. The single most important question that is often asked about a docking algorithm is â€œHow efficiently can the algorithm differentiate between binders and non-binders?â€#157; For the docking algorithm to efficiently differentiate between them, both the conformation search algorithm and the scoring functions must work in tandem at their best capacity. A huge data set of chemical decoys is employed to validate the accuracy, which is spiked with known binders. Chemical decoys are chemical structures that physically resemble known binders but have a different topological arrangements of atoms. The accuracy of the docking algorithm is assessed by its ability to recover known binders from the pool of decoys. However, it is overambitious to expect any docking algorithm to recover all the known binders and none of the decoys. To quantify the accuracy of the docking algorithms and the parameters used, an enrichment factor (EF) [36] is used, which is computed using Eq 2.

$$\;EF=\frac{\frac{ligands_{\left(recovered\right)}}{N_{\left(recovered\right)}}}{\frac{ligands_{\left(total\right)}}{N_{\left(total\right)}}}$$ (2)

Here, *ligands*_(*total*)_ and $ligands_{\left(recovered\right)}$ denote the total number of known binders and binders recovered, respectively, and *N*_(*total*)_ and $N_{\left(recovered\right)}$ denote the total number of decoys and recovered decoys, respectively. From the formula, we can infer that a higher value suggests an effective docking algorithm and parameters thereof. A comprehensive discussion on search algorithms and scoring functions is beyond the scope of this article, and the readers are advised to read the listed papers in the literature [37, 38]. However, we enlist the scoring functions with their succinct descriptions.

#### Force field-based scoring function.

Force fields [39] are mathematical frameworks that describe the potential energy of a molecular system as a function of the positions of its atoms. In other words, they describe chemical information in the numerical information that is more suitable for computations. To do so, various components, each representing a specific type of interaction between atoms. Each component is characterized by its own parameters, such as the strength of the interaction and the range of distances (*r*) over which it is effective. A force field typically consists of:

1. Bonded Interactions
  (a) Bond stretching
  (b) Angle bending
  (c) Torsional interactions
  (d) Improper torsions
2. Non-Bonded Interactions
  (a) Van der Waals forces
  (b) Electrostatics

The bond stretching and angle bending are generally considered hard terms because the energy required to bring about a small change is generally very large. Moreover, to improve the computational speed, these are treated as “fixed” or “constant”. The torsional and improper terms are changed to bring about a conformational change by the conformational search algorithm. Owing to such changes, the non-bonded interactions vary as different conformations lead to different atomic interactions, which is show in Eq 3 as a docking score.

$$E=\sum_{i}\sum_{j}(\frac{A_{ij}}{r_{ij}^{12}}-\frac{B_{ij}}{r_{ij}^{6}}+\frac{q_{i}q_{j}}{\int (r_{ij})r_{ij}})$$ (3)

Where *r*_*ij*_ is the atomic distance between protein atom i and ligand atom j, *A*_*ij*_ and *B*_*ij*_ are vdW repulsive and attractive parameters, respectively. *q*_*i*_ and *q*_*j*_ are atomic partial charges on atoms i and j. The first two terms estimate the van der Waals interactions using Lennard-Jones 12â€"6 potentials, which are commonly used in most force fields. The last term estimates the electrostatic component of the interaction, with solvent effects implicitly defined using the term $\epsilon (r_{ij})$, which is the distance-dependent dielectric constant. It is known that incorporating solvation effects is far from perfect, and without it, the electrostatics will be overestimated for the charged ligands owing to the absence or incorrect dielectric effect seen in the solvated systems. There are several approximations introduced such as Generalised-Born (GB) [40] and Poisson-Boltzmann (PB) models [41, 42], which are popular and computationally efficient methods that estimate solvent effects.

#### Empirical scoring function.

$$\Delta G=\sum_{i}W_{i}\cdot \Delta G_{vdW}+\sum_{j}W_{j}\cdot \Delta G_{ele}+\sum_{k}W_{solv}\cdot \Delta G_{solv}+...$$ (4)

Where $\Delta G_{vdW}$, $\Delta G_{ele}$, and $\Delta G_{solv}$, depict the free energy contribution of van der Waals’, electrostatic, solvation/desolvation, etc. $\sum_{i}W_{i}$, $\sum_{j}W_{j}$, and $\sum_{k}W_{solv}$ are the corresponding coefficients that are adjusted to fit the experimental binding affinity data. The final score is obtained as the sum of all the energy components. Different scoring functions in this class include or exclude certain components, while others use a different fitting method that best fits the binding affinity data.

#### Knowledge-based scoring function.

$$w(r)=-k_{B}T[\frac{\rho (r)}{\rho^{*}(r)}]$$ (5)

Where *w*(*r*) is the final score which is dependent on the atomic distances *r*. *k*_*B*_ is the Boltzmann constant, *T* is the absolute temperature in *K*. ρ(r) is the frequency of protein-ligand atom pairs at distance *r*, and $\rho^{*}(r)$ is the frequency of protein-ligand atom pairs in the reference state at distance *r*. The reference state is generally the frequency that a particular atom pair is observed in the experimental structures. The type of interaction (intermolecular atom pairs) that appears more frequently in the reference state will be scored higher. Therefore, the ratio $\frac{\rho (r)}{\rho^{*}(r)}$ can be considered as a pair distribution function.

#### Consensus scoring function.

$$\Delta G=W_{1}\cdots coringfunction_{1}+W_{2}\cdots coringfunction_{2}+W_{3}\cdots coringfunction_{3}+...$$ (6)

Where *W*_1,2,3,.._ are weights assigned to each of the *scoring function* 1, 2, 3, etc. These weights may not be directly linked to the atomic physicochemical properties. As we have seen so far, each type of scoring function comes with its own set of advantages and disadvantages. There have been many attempts to use more than one type of scoring function simultaneously to improve their overall scoring accuracy. An ideal consensus scoring function will use one or more scoring functions that complement the pros and cons. This is expected to minimise the cons and amplify the pros of each scoring function. However, this is far from reality, and often times, new limitations are observed.

Few questions that frequently arise, especially among the beginners, is *what are generally the units of the docking score?* The units are usually units of energy, expressed as “kcal/mol” or “kJ/mol”. However, it is not unusual to observe scoring functions that yield unitless docking scores. Irrespective, of the units of the docking score, a generally accepted convention is such that a more negative score indicates a better binding score. We would also point out that not always a more negative score indicates “correct” binding conformation (also referred to as “pose”). *Are docking scores obtained from different scoring functions directly comparable?* This is rather difficult to answer holistically because scoring functions could be comparable as they share common philosophy of development or datasets. In our experience, we have always avoided any head-to-head comparison of scores, nevertheless, we compare the rank-ordering given by different scoring functions [43].

## Ten quick tips

### Tip 1: Know your biological target and its functional state

The quality and accuracy of the results obtained from molecular docking, largely depends on the quality of the tertiary structure of the biological target. The structures are resolved using X-ray crystallography, Nuclear Magnetic Resonance (NMR) spectroscopy, and cryogenic electron microscopy (CryoEM). These methods provide with high quality structural data, however, introduce errors such as missing atom information or multiple amino acid rotamers [44, 45]. Furthermore, X-ray crystallographic and CryoEM resolved structures are single snapshot of the receptor structure, while NMR derived structures provide insights in to the dynamics. Hence, it is crucial to evaluate and correct these structural defects before using them for molecular docking. Emphasis should be on the binding site amino acids, their protonation states, and appropriate rotameric states. Furthermore, the receptor’s conformational state or functional states are important. Since most of the experimental structures do not contain this information, use of multiple structures resolved experimentally under different conditions or use MD simulations is highly recommended to include target flexibility. The worldwide Protein Data Bank (wwPDB) is the primary source for receptor structures [46]. In the absence of experimental data, computational methods, like template-based modelling and *ab initio* predictions, can be used to predict the tertiary structures from primary sequences. These theoretical models require thorough validation to ensure accuracy of the predicted tertiary folds. With recent developments in AI-based methods in protein structure prediction, such as AlphaFold [20], high quality structure are readily available in the wwPDB annotated as Computed Structure Models (CSMs).

### Tip 2: Choose the receptor entry in the wwPDB that is closer to the biological problem at hand

It is possible on many occasions that several entries for the same receptor are available on the wwPDB. One must choose based on the conformational state of the receptor that is desired. Then selecting the entry with a good resolution, in our opinion, a resolution below 2.0 Å is a good choice whenever possible. There are other structure quality parameters, apart from the resolution, that provide useful information about the target. The b-factors or beta-factors provide key information on the flexibility of the atoms. It also holds key information on the confidence with which the crystallographers are able to narrow down the atomic positions while fitting the electron density maps, for examples atoms with b-factors ≤40Å^2^ provide good confidence in their assignments. While using the CSMs, predicted local distance difference test (pLDDT), ranges from 0 to 100, depicts a per-residue measure of local confidence that must be carefully examined. pLDDT values ≥70 suggest good confidence in the prediction, while values <50 indicate very low confidence. The users are directed to the Guide to Understanding PDB Data with a section dedicated to CSMs. The choice of receptor should also bear in mind if there exists an entry with a co-crystallised ligand in the binding site of interest. While preparing the receptor, the atomic resolution limitation makes it difficult to locate the position of hydrogens, which must be added in the preparation step. Partial atomic charges are calculated and assigned for individual atoms. The decision to keep important metals, co-factors, and water molecules in the docking site is made at this stage. If they are crucial for ligand binding, they must be retained; otherwise, they can be removed. Such decisions are far more complex than they appear at first sight. To come to a meaningful decision, the computational chemist must be well aware of the biology and functioning of the receptor in question ( **see Tip 1**). Recently, there has been a particular interest in developing tools that systematic evaluate the energetics of displacing water molecules from the binding site by the ligands. In doing so, they are well placed to predict crucial water molecules that may lead to significant entropic gains. The tools such as waterMap [47, 48], WaterDock [49] and SSTmap [50] are available that predicts the energetics of displacing water molecules from the binding site. Deleting co-factors, waters, or metal ions deemed important can lead to meaningless results, even though very high or favourable docking scores are observed.

#### File formats for macromolecules.

1. *PDB (Protein Data Bank):* Stores 3-Dimensional structural data of proteins and nucleic acids.
2. *PQR:* A variant of PDB with atomic charges and radii, often used in electrostatics calculations.
3. *MMCIF/MMTF:* Compressed formats for macromolecular structures, useful for large datasets.

Generally, it is highly recommended to perform energy minimization using a force field compatible with the docking program. This step optimizes all the inconsistencies in the structure or shows warnings on the errors that can be detrimental to the reliability and accuracy of the results.

### Tip 3: Druggability is the key when defining a potential binding site for docking

Docking on a receptor structure is performed in a fixed space, called the binding site. Generally, one must choose the receptor structure from the wwPDB that contains a co-crystallised ligand within the binding site of interest. The search space for docking is defined by selecting amino acid residues 3-6 Å around the ligand. However, we may not always have information on the location of the binding site; in such cases, we can perform a blind docking approach, wherein, the whole receptor surface is defined as the search space. Alternatively, several algorithms are available that can locate potential binding sites, which are ranked according to their likelihood of being a druggable site. Here are a few examples of programs used to locate potential binding sites, SiteMap [51], LIGSITE [52] and Fpocket [53] If the docking program allows target flexibility to accommodate conformational changes induced by the ligand, the number and identity of flexible amino acids and the degree of flexibility are defined.

### Tip 4: Learn the fundamentals of the docking algorithm

There are a plethora of docking programs available (Table 1), some with commercial licenses and others with open-source licenses. Knowing every program to make a choice that best suits you is almost always impossible. However, this is also not necessary as there exists ample literature reporting the use of many of the tools. Irrespective of the choice of the docking program, the general steps will be same; for example, preparation of the receptor ( **see Tip 2**), defining the binding site as search space( **see Tip 3**), preparation of ligands ( **see Tip 5**), specifying the docking parameters, validating the docking procedure, performing the docking calculations, and analysis and dissemination of the results. In a nutshell, the general principles remain unchanged; however, there are differences in ligand format, docking methods, and scoring algorithms that are well-documented in the user manual. We strongly recommend that before using any docking algorithm, the user manual must be consulted to understand each parameter’s importance. The developers present their report on several validation studies as a part of the software development which is a great resource to understand the effect of each parameter on the outcome. A literature survey of the program’s usage will also be covered in the user manual.

**Table 1:** List of common molecular docking software.

Software	License type	Scoring Function	Link to access
AutoDock	Open Source	Force field	AutoDock
AutoDock-Vina	Open Source	Empirical	AutoDock-Vina
DOCK	Open Source (Academic)	Force field	DOCK
GOLD	Commercial	Multiple options	GOLD
FlexX	Commercial	Empirical	FlexX
Glide	Commercial	Empirical	Glide

### Tip 5: Filter and prepare ligands to enhance accuracy and speed

In this case, the small molecules that are docked into a receptor pocket are referred to as ligands. It is not uncommon for the terms “ligands” and “drugs” to be used interchangeably. However, it is important to note that while all drugs are ligands, not all ligands are drugs. Ligands are typically represented as two-dimensional connectivity of atoms, and hence, called a 2D graph. These are converted to three-dimensional representations and energy-minimized for docking. When dealing with a large library of ligands [54] (ranging from millions to billions), an initial filtering process is applied to select drug-like molecules. Various criteria are considered, such as molecular weight, log P, the number of rotatable bonds, and the number of hydrogen bond donors and acceptors. One such filtering rule or criterion named “Lipinski’s Rule of Five” was put forth by Christopher Lipinski [55]. Since the first publication there have been significant amendments to this rule that have improved the ligand filtering criteria [56]. These filtering criteria are applicable for orally administered drugs that do not involve any active or receptor-mediated transport across the bilayer membranes. Nevertheless, these filters may be used to discard chemical structures that are unlikely to be drug or drug-like molecules. **P**an **A**ssay **IN**terference compound **S** (PAINS) [57, 58] is another filter that helps to weed out molecules that tend to show up as actives in all assays and hence must be treated as “False Positives.”

Ligands undergo checks for proper geometry, ensuring that bond distances and angles are reasonable. If necessary, the conformation of the ligands can be minimized to achieve the correct geometry. However, depending on the conformational search algorithm of the docking program, it would warrant the use multiple fixed conformations or allowing the conformations to be generated on the fly. The formers case is relevant for docking programs that inherently treat ligand and receptor rigid bodies, and their user-defined multiple conformations serves as a source of ligand flexibility. These days most of the docking programs are bundled with their own conformational search engine, while user can still instruct which of the torsional degrees are flexible or rigid (*default settings treat all torsions are flexible*).

Ligands containing stereocenters are treated as independent enantiomers. A point to remember, in molecular mechanics the tautomers are treated as different molecules as opposed to quantum mechanics. When one wants to evaluate the binding affinity of tautomers, they must be prepared and docked separately. This is particularly important in evaluating which of the tautomeric states are more suitable for binding. A case study on why tautomeric states should be treated with great care is exemplified by chemical class of biguanides and can be found in ref [59]. Furthermore, the protonation states are selected based on the pH that closely mimics the physiological or experimental conditions. Protonation states can be predicted using software tool like Epik [60, 61], PlayMolecule pKAce [62] or Dimorphite-DL [63]. Table 2 enlists commonly used tools for ligand preparation and format conversion.

**Table 2:** List of Tools for Ligand preparation and format conversion.

Software	License type	Link to access
RDkit	Open Source	RDkit
Auto3D	Open Source	Auto3D
OpenBabel	Open Source	OpenBabel
LigPrep	Commercial	LigPrep
Avogadro	Open Source	Avogadro
MGLTools	Open Source	MGLTools

#### File formats for ligands.

1. *SMILES (Simplified Molecular Input Line Entry System):* A text-based representation of molecular structures. These are 1-Dimensional strings that are suitable for large chemical databases that need to store billions of molecules.
2. *MOL/MOL2:* Describes molecular structures, including 3-Dimensional coordinates and atom types; widely used in docking.
3. *SDF (Structure Data File):* Stores multiple molecules with detailed information like bonds and charges, and various descriptors and other related data.
4. *PDBQT:* Specific to AutoDock and Vina, includes torsional degrees of freedom for ligands. Q denotes charges and T denotes torsional degrees of freedom. This file format can be considered as extension or variant of PDB with additional details on charged and torsions not included in the PDB format.
5. *CML (Chemical Markup Language):* XML-based format for storing chemical information

Since these steps are complex and error-prone to perform, pre-computed libraries from ZINC15 can be downloaded to initiate large-scale screenings [64].

### Tip 6: Compare the docking outcome of known molecules with experimental data to validate the docking protocol

One of the most important, albeit neglected, steps in molecular docking is validation. If the receptor was co-crystallised with a ligand in the receptor site of interest, the protocol must be validated by re-docking it and checking if the experimental binding conformation can be reproduced. However, when there are no bound ligands reported, we advise gathering from the literature a few molecules targeting the receptor in question with experimental binding affinity values reported. After docking these molecules, a rank ordering is carried out to understand if the docking parameters can reproduce the experimental binding affinities in the correct order. We strongly recommend that trying to reproduce the binding affinity values as determined by experimental methods is extremely difficult due to inherent limitations in the scoring functions. These limitations are outlined briefly in the previous section and we also direct the readers towards a comprehensive article for further explanation [65] A large collection of data can be found in the BindingDB or ChemBL. However, it must be noted that these data may contain results from different experimental protocols making a direct comparison difficult.

Another crucial yet often overlooked question is the number of poses that must be generated to ensure more accurate predictions. The number of poses generated in molecular docking varies based on the docking software and the objective of the study. Generally, protocols aim for 10â€"20 poses per ligand (in our group the standard practice is to generate 10 poses minimum) [54] to explore binding conformations effectively. While working with completely new scaffolds, it becomes difficult to establish which docking pose would be more physiologically relevant. In such cases, we recommend evaluating at least two to three diverse poses and then using MD simulations followed by MM-GB/PB-SA methods to estimate the binding affinity (see **Tip 7**)

### Tip 7: Rescore the ligands with independent scoring methods to ensure reliability

It’s important to note that docking scores are mere estimations of the true binding constant (*vide supra*). They rely on the non-covalent interactions between the ligand and receptor and often disregard the entropic component of binding. Therefore, rescoring must be performed with an independent scoring function to ensure reliability. Three post-docking corrections are outlined here:

- Estimating the impact of solvent
- Using consensus scoring, and
- Calculating the absolute or relative free energy of binding.

Solvent plays a crucial role in ligand binding by forming specific interactions with ligands. The strength of electrostatic interactions depends on the dielectric screening effect of the solvent. Some docking algorithms consider solvent effects, while others do not. If solvent effects are not considered, including a solvation correction to the score can enhance the accuracy. MM-GB/SA and MM-PB/SA are well-known methods that can be used as rescoring methods that use implicit solvation correction to the gas phase energies [66]. Consensus scoring, where the top hits from the docking exercise are rescored using different scoring algorithms, has been effective in improving accuracy. Hits that appear at the top of multiple lists are selected for further investigation. Free energy perturbation (FEP) calculations offer a rigorous way to measure the changes in free energy between unbound and bound complexes in the solvent.

### Tip 8: Be consistent with the atomic representations and figures

In addition to advanced rescoring methods, it is also important to visually inspect the docking solution to assess if the protein-ligands show prominent interactions such as hydrogen bonds, salt bridges, water-mediated interactions, cation-π and π-π. Since the results need to be communicated to a wider audience, the colour coding for receptor and ligand atoms must be distinct. The atom colouring and type of interactions must be explained clearly to avoid ambiguity. Additionally, the docking figures should be consistent, and the orientation of the binding poses must clearly illustrate the molecular interactions. This approach will also enable straightforward comparisons of docking images within a congeneric series. Whenever feasible, the docking results for theoretical compounds should be discussed alongside the known molecules, as this will enhance confidence in the findings.

Following are our recommendations while preparing the figures to illustrate the results in either 3-dimensional or 2-dimensional interactions.

1. How to Visualize 3D Interactions?:
  - Use molecular visualization tools like PyMOL [67], Chimera [68], or NGL Viewer [69] to display protein-ligand complexes in 3D.
  - Highlight key interactions such as hydrogen bonds, hydrophobic contacts, and π-stacking.
  - Label interacting residues and measure distances between ligand atoms and receptor residues to provide quantitative insights.
2. How to generate 2D Interaction Diagrams?:
  - Tools like LigPlot+ [70], PoseView [71], and Protein-Ligand Interaction Profiler (PLIP) [72] can create clear 2D diagrams.
  - Display interactions such as hydrogen bonds and hydrophobic contacts with same orientation for intuitive comparison.

### Tip 9: Communicate the results to the experimentalists with a biological and/or chemical viewpoint

The results from molecular docking and other computational studies aim to inspire new experimental research or provide more insights to biological observations. While experimentalists may not be experts in computational methods, they are well-versed in the biological aspects being investigated. It is essential to communicate findings without using technical jargon that could hinder understanding. It is also important to acknowledge that experimentalists are not entirely unfamiliar with computational techniques. When presenting the results, each score should be explained as clearly as possible with its physiological significance. The experimentalists are more accustomed to reading new results with respect to standard data, in the context of molecular docking, the scores for new molecular designs can be explained in comparison to known drugs or reference molecules. This presents the results with the correct context and self-explanatory manner.

### Tip 10: Share input files and docking parameters to comply with FAIR guidelines

There have been significant questions raised on the reproducibility of results reported in the area of computational science [73, 74]. Often times, the use of a proprietary software or data not available in public domain makes it difficult to assess the reliability of the methods, and hence the results. A recent criticism on publishing the AlphaFold3 paper in Nature [75] has renewed the need to adopt policies that make data and methods accessible to all, to validate the findings and thus enhance its scope beyond what was envisioned by the authors. The FAIR (Findability, Accessibility, Interoperability, and Reusability) principles are guidelines established in **2016** to improve data management and sharing in scientific research [76]. We briefly explain each term as follows:

- **Findable:** Data should be easily locatable by humans and machines through unique identifiers, rich metadata, and registration in searchable resources.
- **Accessible:** Data must be retrievable using open, standardized protocols, with metadata remaining accessible even if the data is not.
- **Interoperable:** Data should integrate smoothly with other datasets using shared languages and controlled vocabularies, including references to related data.
- **Reusable:** Data should be well-documented, include clear licensing, and provide provenance information to support transparency and reproducibility.

All the input files, preparation steps, and docking parameters should be made available to the scientific community. This ensures fair dissemination of the results, and those who wish can reproduce the results and build upon their own set of experiments to continue and expand the current work. We also advise that even minor modifications must be reported in the methods. This may appear seemingly small but can be crucial for reproducing the results obtained. This can be indicated in the printed pages of the manuscript but would be more helpful in a repository listed in (Table 3) wherein users can download and test it themselves. Moreover, many of the repositories listed below allow to raise issues whenever users encounter errors or inconsistencies not foreseen by the authors or developers.

**Table 3:** List of repositories for share files in the public domain.

Software	Link to access
Figshare	Click here
Mendeley Data	Click here
Harvard Dataverse	Click here
Dryad Digital Repository	Click here
Open Science Framework	Click here
Zenodo	Click here
GitHub	Click here

## Important terminologies

Following are some important terminologies that are often encountered in structure-based drug design in general and molecular docking in particular:

- ***De novo* design:** De novo drug design involves creating new chemical compounds that meet specific criteria through the use of computational growth algorithms.
- **Ligand efficiency (LE):** A metric that was initially introduced to identify favorable fragments by comparing the average binding energy per atom. It is computed by dividing the free binding energy by the number of heavy atoms (Hydrogens are excluded).
- **Partial atomic charges:** Partial charges indicate where an electron is more or less likely going to be in an orbital. $\delta^{-}$ indicates that an atom is electronegative while $\delta^{+}$ indicates an electropositive atom.
- **Polar Surface Area (PSA):** It refers to the surface associated with hetero-atoms and polar hydrogen atoms within a molecule, while excluding nonpolar elements such as carbon and halogens.
- **Solvent Accessible Surface Area (SASA):** The surface area of a molecule that can directly interact with the solvent. It usually measured by a sphere, called as “probe atom” that runs over the surface of the molecule.

## Summary and take home message

*“* **Garbage-in Garbage-out.***”* Molecular docking is a powerful computational technique that, when employed correctly, can substantially augment experimental research rather than supplant it. It is crucial to recognize that molecular docking merely provides an estimate of binding affinity. Consequently, relying solely on docking outcomes to categorize molecules as agonists or antagonists exceeds the capabilities of all docking methodologies. Even the most sophisticated docking and scoring algorithms are susceptible to limitations and may yield erroneous results. Therefore, it is highly recommended to validate all parameters before using them to identify lead compounds or support medicinal chemistry studies. It is advisable to refrain from comparing docking results obtained from disparate algorithms. Upon the identification of potential hits and the subsequent validation of chemical entities as active by experimentalists, the efficacy of molecular docking studies will undoubtedly lead to robust drug candidates.
